# Modified Artificial Diet for Rearing of Tobacco Budworm, *Helicoverpa armigera*, using the Taguchi Method and Derringer's Desirability Function

**DOI:** 10.1673/031.012.10001

**Published:** 2012-08-25

**Authors:** H. Assemi, M. Rezapanah, R. Vafaei-Shoushtari, A. Mehrvar

**Affiliations:** ^1^ Department of Agricultural Entomology, Arak Branch, Islamic Azad university, Arak, Iran; ^2^ Center of Excellence for Organic Agriculture and Insect Virology Laboratory, Biocontrol Department, Iranian Research Institute of Plant Protection, Iran; ^3^ Department of Plant Protection, Azarbaijan Shahid Madani University-5375171379, East-Azarbaijan, Tabriz, Iran

**Keywords:** mass rearing, parameter optimization

## Abstract

With the aim to improve the mass rearing feasibility of tobacco budworm, *Helicoverpa armigera* Hübner (Lepidoptera: Noctuidae), design of experimental methodology using Taguchi orthogonal array was applied. To do so, the effect of 16 ingredients of an artificial diet including bean, wheat germ powder, Nipagin, ascorbic acid, formaldehyde, oil, agar, distilled water, ascorbate, yeast, chloramphenicol, benomyl, penicillin, temperature, humidity, and container size on some biological characteristics of *H. armigera* was evaluated. The selected 16 factors were considered at two levels (32 experiments) in the experimental design. Among the selected factors, penicillin, container size, formaldehyde, chloramphenicol, wheat germ powder, and agar showed significant effect on the mass rearing performance. Derringer's desirability function was used for simultaneous optimization of mass rearing of tobacco budworm, *H. armigera*, on a modified artificial diet. Derived optimum operating conditions obtained by Derringer's desirability function and Taguchi methodology decreased larval period from 19 to 15.5 days (18.42 % improvement), decreased the pupal period from 12.29 to 11 days (10.49 % improvement), increased the longevity of adults from 14.51 to 21 days (44.72 % improvement), increased the number of eggs/female from 211.21 to 260, and increased egg hatchability from 54.2% to 72% (32.84 % improvement). The proposed method facilitated a systematic mathematical approach with a few well-defined experimental sets.

## Introduction

*Helicoverpa armigera* (Hübner) (Lepidoptera: Noctuidae) is a major pest affecting a wide range of economically important plants, including field and horticultural crops in Iran (Naseri et al. 2011) and throughout the world (Jallow et al. 2004; Reddy et al. 2004; Mironidis and Savopoulou- Soultani 2008; Yu et al. 2008). In northern Iran, tobacco plants are subjected to attack by several insect pests, the most serious of which is the budworm, *H. armigera.* Recently, it has been observed in increasing numbers. In tobacco cultivated fields, *H. armigera* predominantly damages young leaves and bolls, causing whole leaves to be destroyed during the crop's reproductive stage. The destruction of the leaves results in considerable loss of yield at harvest.

It is important to be able to economically rear important insects in order to study their life history, behavior, feeding habits, and susceptibility and resistance to chemical pesticides and biological control agents such as insect viruses (Rezapanah et al. 2008). Rearing insects on artificial diets is an expensive process, and is difficult for developing countries where sufficient research funds are unavailable. As a result, the economic threats imposed by insect pests on agriculture will be poorly studied (Ahmed 1983).

Despite large expenditures for development of mass-rearing/artificial diet-based technology over the past five decades, successful commercialization of biological control systems based on such technology has been sparse. Also, many of the shortcomings of diet development are attributable to a failure to recognize the tremendous difficulty in the problems of diet and rearing system development(Cohen 2003). Although diets are incredibly dynamic and complex mixes of multiple interactive functional components, diet development is often treated in the most simplistic manner (Cohen 2003). Cohen (2003) has explained that there are four equally important functional domains of diets: nutritional value, sensory qualities, availability, and stability. However, diet researchers often pay exclusive attention to the nutritional functions of diet components, without considering phagostimulation, textural factors (hydrocolloid characteristics, particle size, particle shape, viscosity, and other rheological matters), meta-nutritional antioxidant functions, anti-nutrients, bioavailability, water activity, antimicrobial potential, over-all stability, microbial deterioration, and numerous other features that must be juggled skillfully to achieve development of fully functional, successful diets (Cohen 2003). He has also explained that a most unfortunate confusion has developed regarding the terms “insect nutrition” and “insect dietetics,” with an even more unfortunate tacit assumption that nutrition is a “real science,” and dietetics an extension of fast food cooking. As a result of this confusion, and the prejudice against dietetic inquiries, researchers neglect most of the key issues that would explain how diets and certain diet components work or fail to work (Cohen 2003).

So far, various artificial diets have been developed and proposed for the maintenance and continuous rearing of economically important insects (Ahmed et al. 1998; Cohen, 2001; Castane and Zapata 2005). Although there is some success in efforts to rear successive generations of these insects entirely on an artificial diet, in many cases there is loss of both fitness and reproductive potential, which causes longer developmental period and lower fecundity rate (Coudron et al. 2002). As a result, the cost-saving ratio is diminished. For those who work on life and fecundity tables of insects, it is important to know on what type of diet the insect has been reared. This factor affects the accuracy of biological characteristics of the insect, e.g. growth, viability, reproduction and population density, and consequently the accuracy of the life table (Bellows et al. 1992). Moreover, the shifting emphasis in insect control, using biological entities such as natural enemies and insect pathogens (bacteria, viruses and fungi), has increased the demand for constant reliable sources of such insects.

Laboratory-reared larvae can be used for the study of insect pathogens, plant resistance factors, effects of insecticides and radiation on fecundity and growth, as and insect life cycle (Ahmed et al. 1998). Beck et al. (1949) reported the first successful aseptic rearing of phytophagous moth, the European corn borer, *Ostrinia nubilalis* Hübner (Lepidoptera: Pyralidae), on a meridic diet. Ishii and Urushibara (1954) were able to rear the rice stem borer, *Chilo suppersalis* Walker (Lepidoptera: Crambidae) on a modification of the diet reported by Beck et al. (1949). Vanderzant and Reiser (1956) were the first to formulate a meridic diet for the pink bollworm, *Pectinophora gossypiella* Saunders (Lepidoptera: Gelechiidae), without a plant adjuvant. Later, Adkinsson et al. (1960) developed a satisfactory artificial medium supplemented with wheat germ for rearing pink bollworm. The wheat germ medium has been used to rear several species of plant-feeding lepidopterans, including the bollworm *Helicoverpa zea* Boddie (Lepidoptera: Noctuidae) (Vanderzant et al., 1962); the cabbage looper, *Trichoplusia ni* Hübner (Lepidoptera: Noctuidae) (Getzin, 1962); the white large butterfly, *Pieris brassicae* Linnaeus (Lepidoptera: Pieridae) (David, 1965); and the codling moth, *Carpocapsa pomonella* Linnaeus (Lepidoptera: Tortricide) (Rock et al., 1964).

Ahmed et al. (1998) reported that agar is a vital ingredient for insect-rearing diet, and is acquired from marine algae such as the *Gracilaria* and *Geladiella* species (Nene 1996). Abbasi et al. (2007) showed that when larval stage *H. armigera* were fed with a tapioca-based artificial diet, larval and pupal developmental period, percent pupating, pupal weight, emergence rate of male and female, longevity, fecundity, and hatching were non-significantly different from those of the control agar-based artificial diet.

Different models can be used for various design of experiments. For example, Hare (1992) used simple models to study trace metal contamination, and its effect on both distribution and abundance of aquatic insects. He found choosing a predictive model will facilitate the relationship between nature and laboratory data.

Lapoinite et al. (2010) employed response surface methodology (RSM) in order to determine the effects of proportionality and amount of diet ingredients on larval survival, weight gain, and development rate in *Diaprepes abbreviatus* (L.). In his research, all measured larval responses led to the conclusion that cottonseed meal is the only major nutritive component, in addition to standard vitamin and salt mixes, required for a successful artificial diet for rearing larvae of *D. abbreviatus* to pupation. The response surface models demonstrated that larval survival to pupation and larval weight gain for *D. abbreviatus* are responses that can be independently manipulated. Diets that produce large insects do not necessarily produce the greatest number of insects (Lapointe et al. 2008). Their experience in previous experimentation (Lapointe et al. 2008) was that survival of pupae to adult emergence was invariably close to 100%.

There are many statistical procedures used to design and analyze an experiment, but the most frequently used approach is a full factorial experiment. However, for full factorial experiments, there are 2 possible combinations that must be tested (f = the number of factors each at two levels). Therefore, it is very time-consuming when there are many factors involved (Ross, 1989). In order to minimize the number of required tests, fractional factorial experiments were developed. However, a shortcoming of this method is only a few of the total possible combinations are used in order to estimate the effects of main factors, and the effects of some of the interactions.

Taguchi developed a family of fractional factorial matrices that could be utilized in various situations. These matrices reduce the number of experiments, while providing valuable conclusions. The conclusions can be associated with a statistical level of confidence (Ryan 1988; Ross 1989; Peace 1992). The major steps of implementing the Taguchi method are: (1) identify the factors/interactions, (2) identify the levels of each factor, (3) select an appropriate orthogonal array, (4) assign the factors/interactions to columns of the orthogonal array, (5) conduct the experiments, (6) analyze the data and determine the optimal levels, and (7) conduct the confirmation experiment. The Taguchi method uses the signal to noise ratio to measure the quality characteristics deviating from the desired value. The signal to noise ratios differ according to the type of characteristics. The “smaller the better” and “ the bigger the better” responses are considered, aiming to minimize or maximize responses which are calculated using the following equations (Akhyar et al. 2008):

The smaller the better response category:


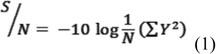


The bigger the better response category:


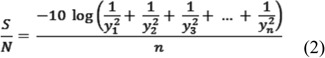


where y_i_ is the characteristic properties: mass rearing, n is the replication number of the experiments.

In our study, we tried to determine the impact of the most critical ingredients in an artificial diet (such as agar, bean, ascorbic acid, ascorbate, formaldehyde, yeast, wheat germ powder, etc.), all of which have significant influence on the mass rearing of *H. armigera.* One strategy used in order to achieve the most effective performance is the optimization of operating parameters to create an optimum mass rearing of *H. armigera.* In order to establish better conditions by relating all the factors considered, numerous experiments need to be carried out with all the possible parameter combinations, which is not practical. Conventional experimental procedures involve altering of one factor at a time, and keeping all other factors constant, and assessing the impact of the individual factors. This procedure is, however, time consuming, requires more experimental sets, and is unable to provide information about how the individual factors interact with each other. Design of experiments is an approach that helps to gain information about the optimized levels by taking a large number of variables into account. In our study, by performing Taguchi's method, and by making some modifications on a series of parameters, we were able to define the best combination of parameters for mass rearing feasibility of the *H. armigera* larvae.

## Materials and Methods

A colony of *H. armigera* was raised from two pairs of adults collected from a tobacco field at Tirtash Research and Education Center, Behshahr, Iran, and transferred to the laboratory. Then, it was reared on the agarbased diet (Ahmed et al. 1998), and used as a control. Experiments were conducted in the controlled laboratory conditions of 65 ± 5% RH, 27 ± 2 °C, and 14:10 L:D. A homogenous stock of different larval instars, eggs, pupae, and adults were obtained from F1 generation for testing. The experiments were carried out with 16 factors at two levels, as shown in Table 1. The ingredients used in this study were selected according to Teakle and Jensen's work (1985) with an increase of about 14.28–50.00% in the quantities of all the components.

The dry ingredients of the diet were weighed carefully, and kept in separate containers. The wet ingredients were measured, and also kept in separate containers. The entire quantity of agar was suspended and brought to a boil. The total quantity of bean was added to the boiled agar. Then, all the dry and wet ingredients were added to this mixture. The prepared diet was then poured into the desired number of sterilized Petri plates, and allowed to cool and harden.

### Rearing procedures

**a) Petri plate for Individual larval development.** In our study, two standard-sized Petri plates (6 and 9 cm diameter, 1cm high) were used in the Taguchi design. Initially, these sterilized dishes containing food were infested with 20–30 newly hatched first instar larvae, with the help of a sterilized camel hair brush. Then, to prevent larval cannibalism, the larvae were divided into 10 sets, each comprising 30–50 dishes (one larva/dish).

**b) Adult emergence.** The larvae that were pupated in Petri plates were taken out and placed in the plastic dishes with a thin layer of soil at the bottom, and covered with muslin cloth. After emergence, the adults were collected individually into oviposition dishes. The emerged adults were checked and removed daily.

**c) Mating- oviposition cage.** 50 boxes (8 × 15 × 20 cm) made of acrylic sheet were fabricated into an oviposition cage. A pair of adults were placed in each oviposition cage. These cages were covered with muslin cloth for ventilation. In order to provide food for the gravid females, several cotton wool pads, soaked in 10% sucrose solution, were placed on muslin cloth. The eggs deposited on the layers of the cotton wool oviposition pads were checked and removed daily, and kept in polyethylene bags for incubation and hatching.

**d) Egg incubation.** The eggs were incubated at two temperatures & humidity, i.e., 20 °C and 25 °C, 60 % and 70% RH. The hatched larvae were then transferred into Petri plates containing their diet. The percent of egg hatching was recorded daily.

### Statistical analysis

In our study, the obtained experimental data was processed using Qualitek-4 (Nutek Inc. Bloomfield Hills, MI) software (Venkata-Mohan et al. 2007). Also, an analysis of variance (ANOVA) was applied to the data in order to conduct an analysis of the relative importance of each factor more systemically.

## Results

### Mass rearing by employing Taguchi method

In Taguchi method, if the factors and interactions are more than 15, a possible matrix is a thirty two-trial orthogonal array, which is labeled as L32 matrix (Growley 1988; White 1991; Speidell et al. 1994). As seen in Table 2, mass rearing experiments were performed using selected 32 experimental trials, in combination of 16 factors at 2 levels. All sets of experiments, as per assigned factors, and the results obtained from signal to noise ratio for mass rearing using equations 1 and 2 in the case of “the bigger and the smaller, the better ” response categories, are shown in Table 2. As seen in Table 2, some responses, such as average larval period, larval mortality, average pupal period, and pupal mortality, were supposed to be minimized, and the other responses, such as longevity of adults, the number of eggs laid, and egg hatchability, were considered to be maximized. Also, the raw data for average value of signal to noise ratio for each parameter at two levels are given in Table 3. The difference between values at levels 2 and 1 (L2–L1) of each factor indicates the relative influence of the effect. The larger the difference, the stronger the influence. The negative value has been ignored in assessing the main effect as the placement order of levels assigns either positive or negative values (Venkatan-Mohan et al. 2007). ANOVA results for signal to noise ratio data with response characteristics are given in Table 4. The main objective of ANOVA is to extract from the results how much variation each factor causes relative to the result. The larger the variance of each factor, the stronger the influences on the results. According to the results of ANOVA (Table 4), the relative influences of these factors on “the bigger the better” response categories are in the order of: temperature > penicillin > ascorbate > Nipagin > formaldehyde > agar > oil > yeast > distilled water > ascorbic acid > humidity > container size > bean > wheat germ powder > chloramphenicol > benomyl and in the case of “the smaller the better” responses are in order of yeast > ascorbic acid > ascorbate > container size > benomyl > formaldehyde > penicillin > wheat germ powder > water sterile > agar > oil > bean > humidity > Nipagin > temperature > chloramphenicol.

### Optimum levels and estimation of optimum response characteristics

Table 5 indicates that, in the case of “the bigger the better” responses, the first level of agar, bean, ascorbate, yeast, formaldehyde, distilled water, chloramphenicol, wheat germ powder, penicillin, humidity, container size, and the second level of other factors has a large signal to noise ratio (28.40. In the case of “the smaller the better” response categories, the first level of bean, ascorbic acid, ascorbate, yeast, formaldehyde, distilled water, Nipagin, benomyl, temperature, humidity, and the second level of other factors has a small signal to noise ratio (-20.00). For simultaneous optimization of the two different sets of responses (the smaller, the better, and the bigger, the better) partial desirability (d.) and global desirability (D) functions were used, and are described in the following section.

### Partial Desirability (d.) and Global Desirability (D) Functions

The procedure followed in this work for simultaneous optimization of two different sets of responses (the smaller, the better, and the bigger, the better) is a method developed by Derringer and Suich (1980). The method involves transformation of each predicted response, â, to a dimensionless partial desirability function, d., which includes the researcher's priorities and desires when building the optimization procedure. One or two-sided functions are used, depending on whether each of the responses has to be maximized or minimized, or has an allotted target value. If the response i is to be maximized, the quantity d. is defined as:


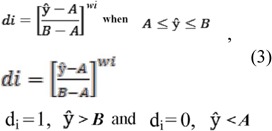


Likewise, d_i_ can be defined when the response is to be minimized, or if there is a target value for the response. In Eq. (3), A and B are, respectively, the lowest and the highest values obtained for the response i, and W_i_ is the weight, d_i_ ranges between 0, for a completely undesired response, and 1, for a fully desired response. In both cases, d_i_ will vary non-linearly while approaching the desired value. But with a weight of 1, d_i_ varies linearly. In our work, we chose weights equal to 1 for all six responses. The partial desirability functions are then combined into a single composite response, the so-called global desirability function D, defined as the geometric mean of the different d_i_-values:


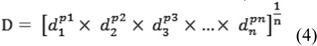


A value of D different from zero implies that all responses are in a desirable range simultaneously, and, consequently, for a value of D close to 1, the combination of the different criteria is globally optimum, so the response values are near the target values. In Eq. (4), pi is the relative importance assigned to the response i. The relative importance of pi is a comparative scale for weighing each of the resulting d_i_ in the overall desirability product, and it varies from the least important (pi = 1) to the most important (pi =5). It is noteworthy that the outcome of the overall desirability D depends on the pi value that offers users flexibility in the definition of desirability functions. According to Table 6, in row 1, *Y_min_* (average larval period, larval mortality, average pupal period and pupal mortality) and *Y_max_* (longevity of adults, the number of eggs laid and percent hatched of eggs) were measured 9.87 and 94.96, respectively. Also, according to equations 3 and 4, d*min*, d*max* and D were defined 0.1, 0.73 and 0.14, respectively. Then, the obtained global desirability functions (D) are used as new responses of Taguchi design, and the other steps of analysis were again carried out by Qualitek-4 software. In this case, predicted results are shown in Table 7. So, It can be concluded that in simultaneous optimization of two different response categories (the smaller, the better, and the bigger, the better), the first level of container size, yeast, oil, chloramphenicol, Nipagin, benomyl, penicillin, agar, and the second level of the other factors have important roles.

According to the final analysis of variance (Table 8), the F—ratio indicates the variation in the response can be explained by the regression. The *p* value is used to estimate whether *F* is large enough to indicate statistical significance. If *p* value is lower than 0.05, it indicates the factor is statistically significant (Segurola et al. 1999). The larger the variance of each factor, the stronger the influence on the results. Hence, from the results of the final ANOVA displayed in Table 8, the order of relative influence of these selected factors on yield of mass rearing in the case of simultaneous optimization of two different set of responses (the smaller, the better, and the bigger, the better) are as follows: penicillin > bean > container size > formaldehyde > agar > ascorbate > chloramphenicol > wheat germ powder > benomyl > distilled water > temperature > humidity > oil > Nipagin > yeast > ascorbic acid. As seen in Table 8, the results showed that, in comparison with other factors, penicillin, bean, container size, formaldehyde, agar, and ascorbate are significant (*p* value is lower than 0.05). For further investigation, six final significant factors (with P value less than 0.05) in our final artificial diet formula (Table 8) were chosen as a control. Then, a new ANOVA table was provided, with these six main factors, three levels (high, medium and low) and, keeping the other factors constant (Table 9, 10). As shown in Table 10, these 6 factors were meaningful at these three levels as well (p< 0.05). Moreover, in order to be sure of the meaningfulness of the primary selected two levels, the Pair t-test was used. In our test, when t-calculated > t- critical, it indicates that there is a significant difference between the levels. Table 11 expresses this claim. Eventually, it is concluded that, based on Tables 8, 10 and 11, these results are in good agreement with each other. In other words, the main factors are the meaningful factors, and there is a significant difference between the selected levels.

**Figure 1.**
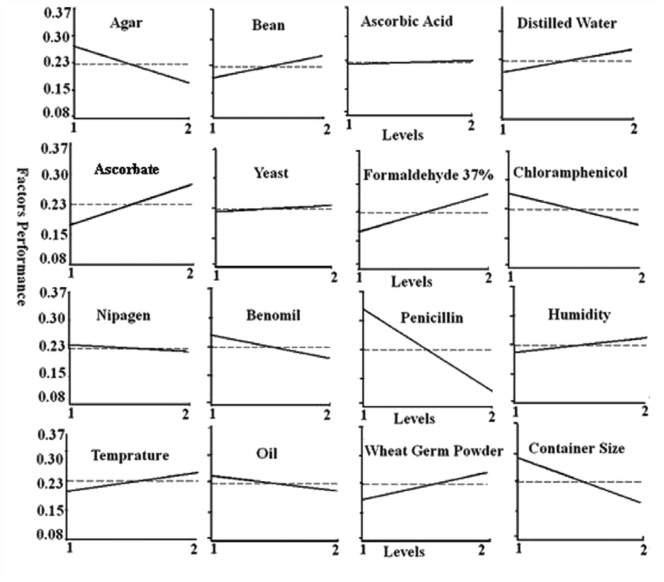
Plots of Individual factors performance at different levels for optimization of modified artificial diet. High quality figures are available online.

The response curves for the individual effects of parameters on average value of responses are given in Figure 1. In Figure 1, an increase in the levels of factors such as bean, ascorbate, formaldehyde, distilled water, wheat germ powder, humidity, and temperature resulted in an increase in the response values. This is discussed further below.

Two conformation experiments were performed at selected optimal levels of the process parameter. The predicted result obtained for the final artiticial diet is D=0.789, with a confidence level of ± 0.148 via Taguchi method. It means that if our confimatory experiment (µ) is 0.641 < µ > 0.937, we can conclude that predicted and confirmatory experiment results are in agreement with each other. In our study, confirmatory experiment was obtained µ=0.916. Note that this result is valid within the specified range.Table 12 shows the comparison of confirmatory experiments and predicted values of different objective functions under the optimum conditions, and it can be concluded that they are in good agreement with each other.

## Discussion

This investigation revealed that using Taguchi and Derringer's Desirability Function approach facilitated the process of optimization by understanding the role of factors involved in the ingredients for the preparation of diet. The final results showed, in comparison with other parameters, penicillin (Level 1 , 2 g), bean (Level 2 , 305 g), container size (Level 1; 6×1 cm), formaldehyde (Level 2; 1 cc), agar (Level 1; 14 g), and ascorbate (Level 2; 5 g) were the most effective factors for the yield of mass rearing (Table 8). An increase in the amount of bean, ascorbate, formaldehyde 37%, and wheat germ powder in the artificial diet resulted in higher mass rearing (Figure 1), because these factors acted as nutrients for insects, and infection reducers for diet.

The results showed a significant decrease in yield of mass rearing with increasing agar, penicillin, and container size. The maximum effect of penicillin for reduction of fungal contamination was obtained at level 1 (2 g), whereas subsequent increases in this factor resulted in the reverse effect due to mortality of larvae. Small size of container can provide desired growth of insect, and even decreased fungal and bacterial contaminations.

According to Figure 1, the highest yield of mass rearing was obtained at the smaller amount of agar (level 1, 14 g), which is in agreement with the results of other studies on artificial diet showing its economical usefulness for mass rearing (Shorey and Hall 1965; Ahmed 1983; Abbasi et al. 2007). In our study other factors had no significant effects on optimization of production. Table 8 shows that, in comparison with other factors, penicillin, bean, container size, formaldehyde, agar, and ascorbate are significant (*p* < 0.05), and we should be careful about amounts of these components. However, it does not mean that the other factors were non-influential; rather, all the factors together help to create the best modified artificial diet. Hence, it can be concluded that final artificial diet in first order are penicillin (Level 1; 2 g), bean (Level 2; 305 g), container size (Level 1; 6×1 cm), formaldehyde (Level 2; 1 cc), agar (Level 1; 14 g) and ascorbate (Level 2; 5 g) that were significant factors, following chloramphenicol (Level 1; 2 cc), wheat germ powder (Level 2; 45 g), benomyl (Level 1; 3 g), distilled water (Level 2; 1000 cc), temperature (Level 2; 25 ^°^C), humidity (Level 2, 70%), oil (Level 1; 5 cc), Nipagin (Level 1; 3 g), yeast (Level 2; 50 g) and ascorbic acid (Level 2; 2.2 g).

**Figure 2.**
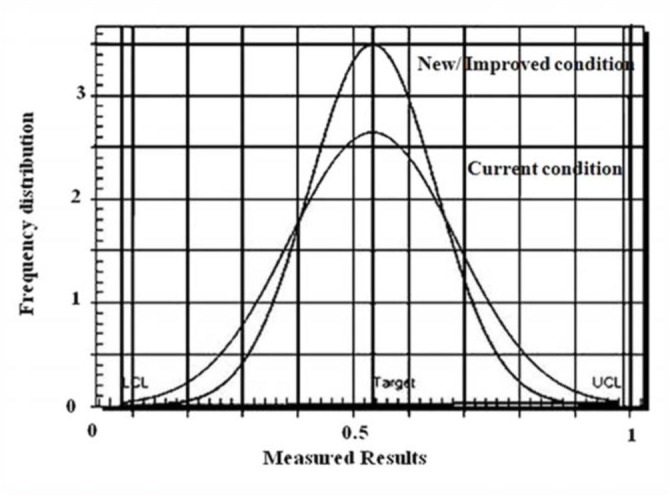
Performance distribution of current (non- optimum) and new/improved (optimum) conditions. High quality figures are available online.

Moreover, the experiments carried out under optimized conditions showed that growth was enhanced. The process variation at improved and current conditions, with the function of frequency distribution, is shown in Figure 2. It can be concluded that a substantial increase in the frequency distribution was observed with the optimal factors. Therefore, it can be concluded that our design was successful.

Derived optimum operating conditions obtained by the Taguchi methodology and Deringer's desirability functions showed effective yield of mass rearing of tobacco budworm so that the larval and pupal periods decreased from 19 to 15.5 days (over all 18.42% improvement), and from 12.29 to 11 days (over all 10.49 % improvement), respectively at 20–25 °C. Under the same conditions, however, Ahmed et al (1998) reported that the average larval and pupal periods were 22.9 and 17.1 days. In this study, the longevity of adult was enhanced from 14.51 to 21 days (over all 44.72 % improvement). The number of eggs/female increased from 211.21 to 260 eggs and egg hatchability increased from 54.2 to 72% (over all 32.84 % improvement).

Compared with what Abbasi et al. (2007) found, our results show the impact of the combined ingredients of different levels on longevity of adult, number of eggs/ female, and egg hatchability, which were 15.03 days, 201%, and 59.01%, respectively. These results are in agreement with Gelman et al. (2001). They found out the highest egg hatchability for leaf-fed beetles fed the Lettuce diet (70.0 ± 8.0%), and lowest for beetles fed the Potato diet (35.4 ± 3.6%). Also, Hamed and Nadeem (2008) studied seven artificial diets in laboratory conditions. They prepared a diet using basic ingredients such as flour of chickpea, mungbean, soybean, wheat, maize, cotton seed, and water chestnut. They reported that larval duration of *H. armigera* was shortest (14.5 days) in the chickpea flour diet, but was longest (42.8 days) in the water chestnut flour diet. In other diets, larval duration was 15.3, 15.5, 15.6, 16.0, 16.5 and 21.3 days on mungbean, soybean, chickpea leaves and pods (control), wheat, maize, and cotton seed, respectively. It was concluded that larval duration (15.5 days) in our study was close to larval duration of soybean, chickpea leaves and pods (control), wheat, maize diets in Hamed and Nadeem's (2008) study. Average larval and pupal mortality in our findings were 4.4% and 7.7%, respectively. Hamed and Nadeem (2008) suggested that the minimum mortality was on chickpea flour (1.1%), while maximum mortality was on cotton seed flour (21.0%). Based on our finding, pupal duration (11.0 days) was equal to the chickpea flour diet investigated by Hamed and Nadeem (2008).

In conclusion, the Taguchi optimization methodology provided a systematic and efficient mathematical approach to understanding complex processes for optimization of mass rearing parameters with only a few well-defined experimental sets. Hence, this diet is recommended as a cost-effective production for rearing of *H. armiger.* Note that our findings from this work are relevant to the two test levels used, and could give different results at other test levels, or at greater extremes than tested in this experiment.

## Figures and Tables

**Table 1.**
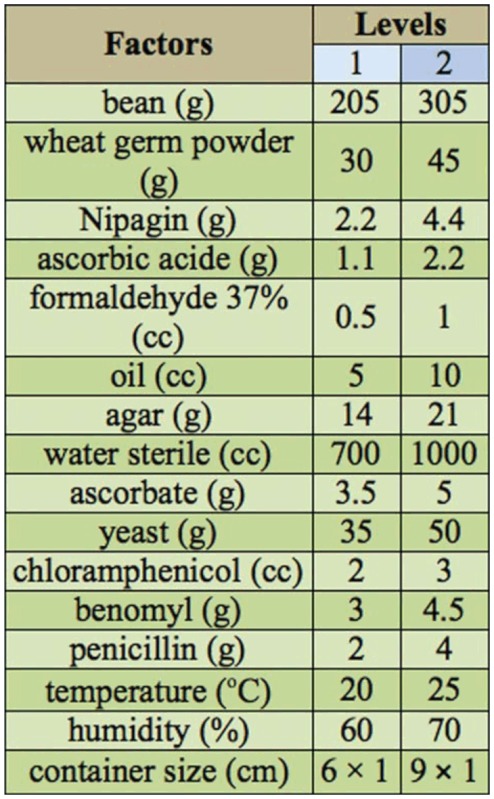
Factors and their levels chosen for optimization of modified artificial diet using Taguchi method.

**Table 2.**
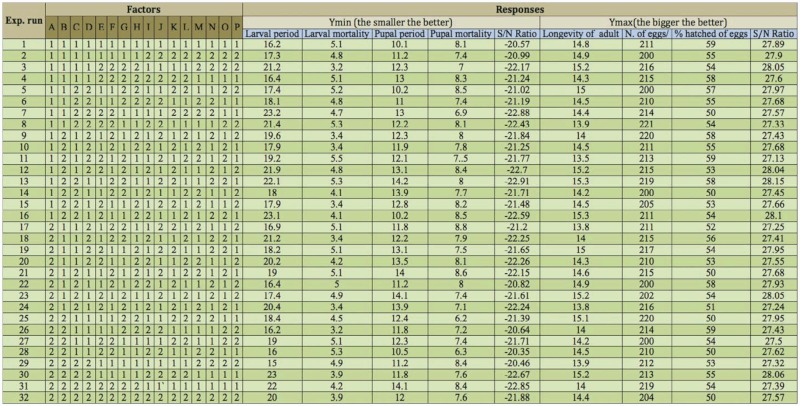
Column assignment for various factors in the Taguchi's L_32_ orthogonal array (OA) and experimental values for optimization of modified artificial diet.

**Table 3.**
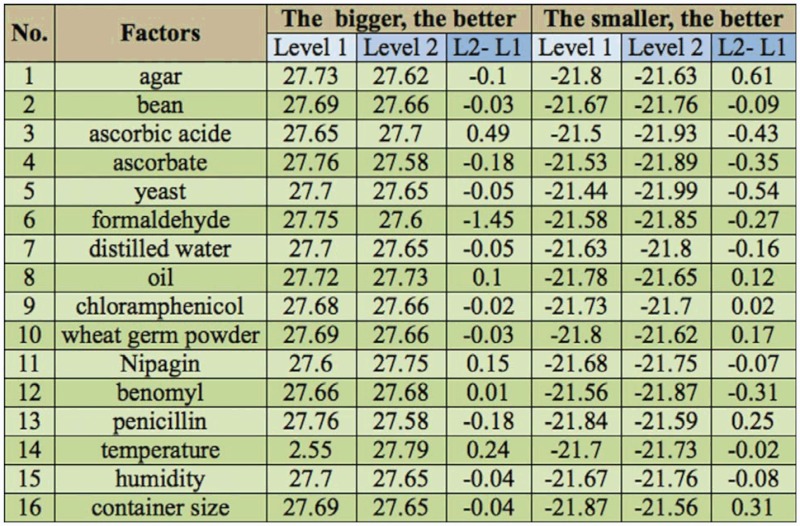
Response (average value of S/N ratio) for Taguchi analysis for “the bigger, the better responses” (average longevity of adults, the number of eggs laid and egg hatchability) and “the smaller the better responses” (average larval period, larval mortality, average pupal period and pupal mortality).

**Table 4.**
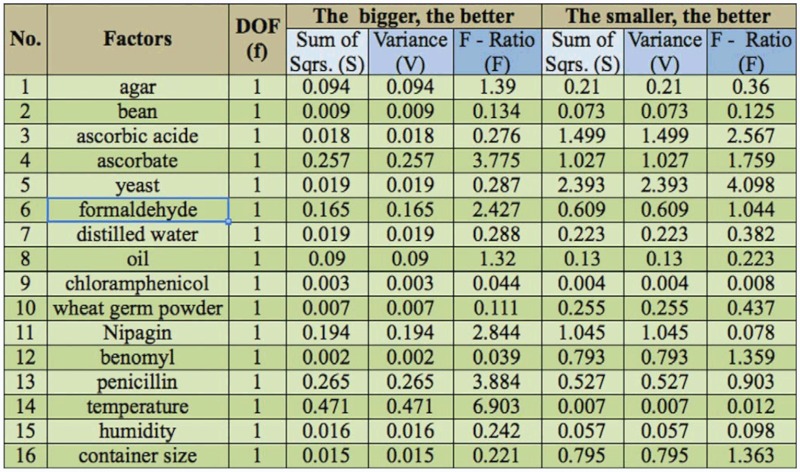
Analysis of variance (ANOVA) for the “bigger, the better responses” (average longevity of adults, the number of eggs laid and egg hatchability) and “the smaller the better responses” (average larval period, larval mortality, average pupal period and pupal mortality).

**Table 5.**
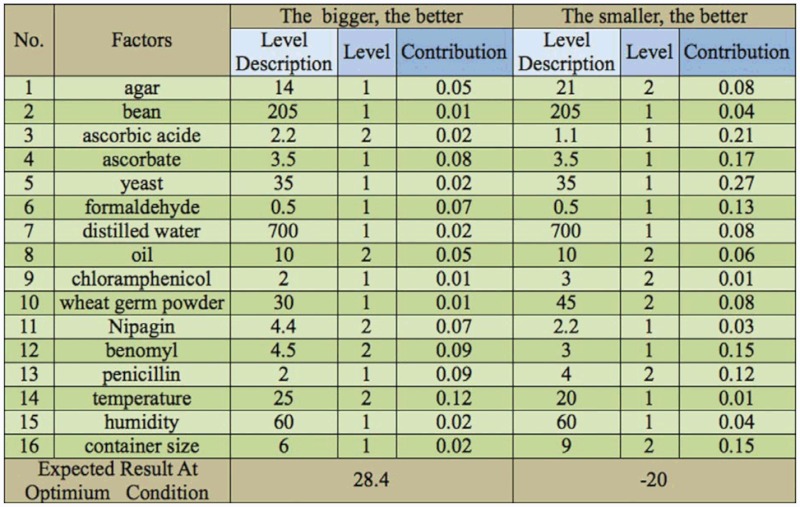
Predicted Optimium Conditions and Contribution for four values of mass rearing using “the bigger, the better” responses (average longevity of adults, the number of eggs laid and egg hatchability) and the “smaller the better” responses (average larval period, larval mortality, average pupal period and pupal mortality) via Taguchi method.

**Table 6.**
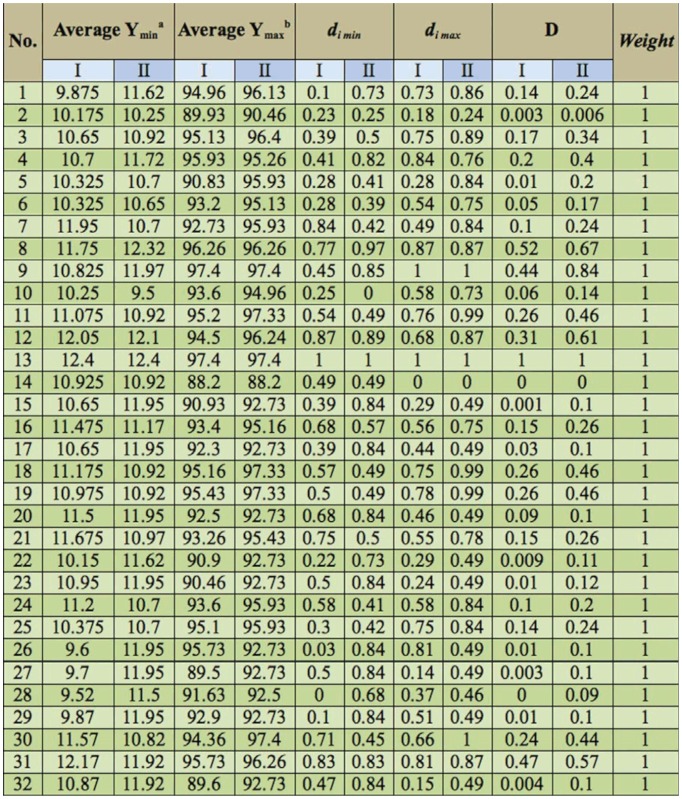
Partial (d.) and global (D) desirability Functions for analysis of seven values of mass rearing (average longevity of adults, the number of eggs laid, egg hatchability, larval period, larval mortality, average pupal period and pupal mortality).

**Table 7.**
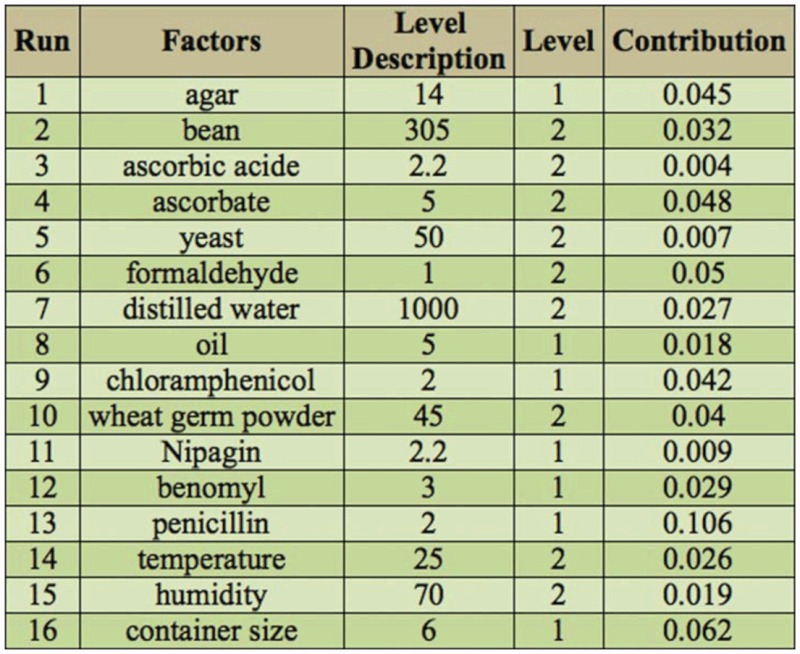
Final predicted results in the case of simultaneous optimization of two different sets of responses (the bigger, the better) using Derringer's desirability function and Taguchi method.

**Table 8.**
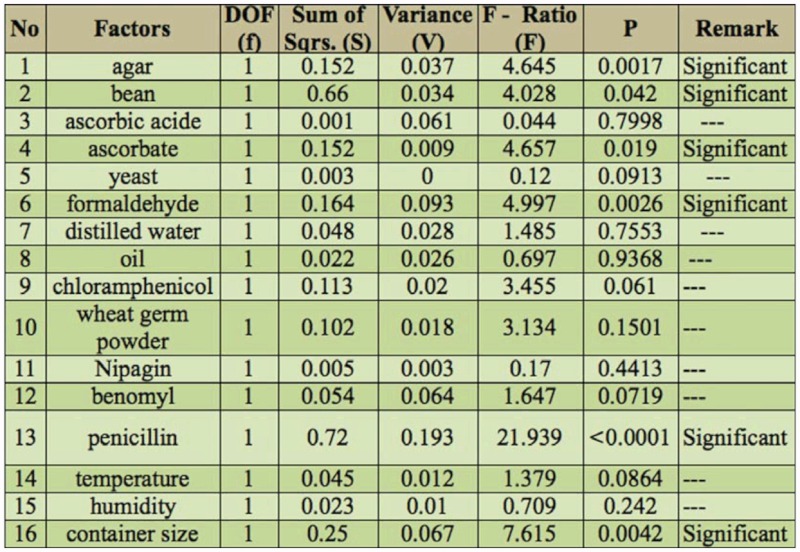
Final Analysis of variance (ANOVA) for optimization of modified artificial diet.

**Table 9.**
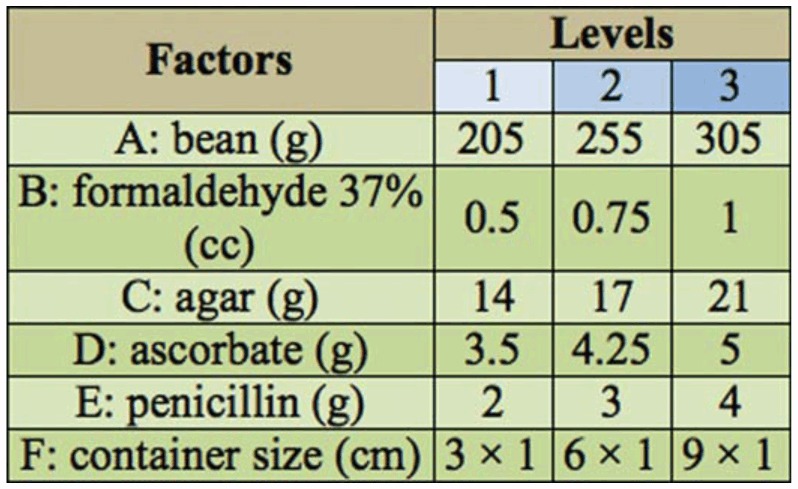
Six main factors with their high, medium and low levels chosen for further investigation of factors significancy.

**Table 10.**
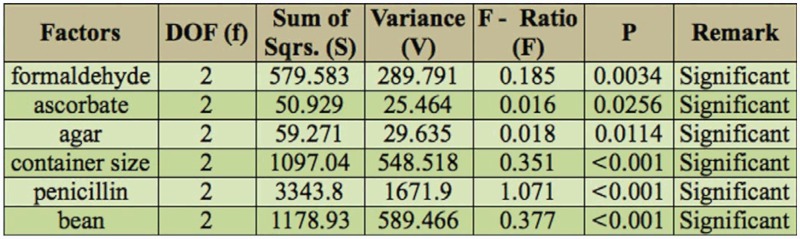
ANOVA table for six main factors with their high, medium and low levels.

**Table 11.**
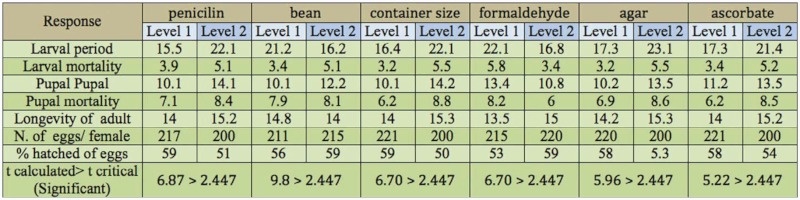
Paired t-test for six main factors with two levels chosen for further investigation of factors significancy.

**Table 12.**
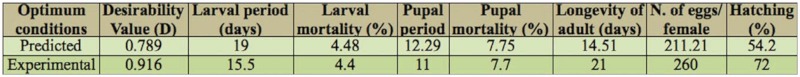
Comparison of confirmatory experiments and predicted values of different objective functions under the optimum conditions.
